# Unknown, so also unvalued? Blood donation awareness and attitudes of potential donors of Dutch and African descent

**DOI:** 10.1111/vox.13029

**Published:** 2020-11-07

**Authors:** Elisabeth F. Klinkenberg, Mirjam P. Fransen, Wim L.A.M. de Kort, Elisabeth M.J. Huis in ’t Veld, Julia C.M. van Weert

**Affiliations:** ^1^ Department of Donor Medicine Research Sanquin Research Amsterdam The Netherlands; ^2^ Department of Public Health Amsterdam UMC Amsterdam Public Health research institute University of Amsterdam Amsterdam The Netherlands; ^3^ Department of Cognitive Science & Artificial Intelligence Tilburg University Tilburg The Netherlands; ^4^ Amsterdam School of Communication Research / ASCoR Department of Communication Science University of Amsterdam Amsterdam The Netherlands

**Keywords:** donor motivation, donor recruitment, donors

## Abstract

**Background and objectives:**

Many Western countries face a shortage of African blood donors, while their specific blood groups are needed to transfuse chronic transfusion patients of similar ethnic background. Blood donation awareness and attitudes greatly impact the decision to become a blood donor, but how they are related and differ across ethnic groups is understudied. This study investigated blood donation awareness and attitudes of individuals of Dutch and African descent in the Netherlands.

**Materials and methods:**

Survey data of 257 African and 152 Dutch non‐donors measuring donation awareness (i.e. being familiar with the Dutch blood bank organization and knowing others who donated blood), cognitive (evaluative judgements) and affective (emotional reactions) attitudes were included. *t*‐Tests, chi‐square tests, linear and logistic regressions were conducted to study differences and associations between donation awareness and attitudes.

**Results:**

African individuals were less often aware of the Dutch blood bank organization (43%; p < 0·05) or others who donated blood (51%; p < 0·05) than Dutch individuals (55% and 68%, respectively). African individuals had lower cognitive donation attitudes compared with Dutch individuals (p < 0·001), but no differences were found for affective attitudes (p = 0·55). High donation awareness was associated with higher cognitive (p < 0·001) and affective (p < 0·05) donation attitudes among African minorities, but not among Dutch individuals.

**Conclusion:**

The lower donation awareness and cognitive attitudes of African minorities should be taken into consideration in donor recruitment. Raising awareness through effective communication strategies might be essential in the donor decision making process of this target group.

## Introduction

Blood donors of African descent are greatly underrepresented in many Western high‐income countries [1]. This presents an important healthcare problem as blood groups are different across ethnic groups. Especially, people of Sub‐Saharan African/Black descent have extended antigen‐negative blood type variations almost non‐existent in European/White populations [2]. Frequent blood transfusions are important for treating patients with blood disorders, including inheritable diseases such as sickle cell anaemia and thalassaemia, with an estimated 300 thousand affected newborns each year [3]. These kinds of diseases are more prevalent in people from African descent as compared to people of, for example, European ancestry. It is essential to adequately match the donor and recipient blood groups, especially in chronic transfusion patients, as antibody formation (alloimmunization) can complicate future transfusions, and future mismatches can cause severe transfusion reactions [4, 5]. Yet, preventive antigen matching and preventing alloimmunization is a significant challenge when the blood donor population is non‐diverse [4]. Thus, individuals of African descent are underrepresented in blood donor pools, but are not underrepresented as recipients for multiple blood transfusions [6].

Consequently, an ethnically diverse blood donor pool adequately mirroring the patient population is of great importance. Although various blood collection agencies have been focusing and adjusting campaigns to recruit and retain more African blood donors [6], in the Netherlands recruitment efforts of this group in particular have been small scale, local and incidental, for example at a few cultural events, mosques and universities. As the Netherlands has an estimated 500 000 residents with a sub‐Saharan heritage [7], which is growing because of migration and childbirth, the Dutch national blood collection agency (Sanquin) now also seeks blood donors of African descent to better match with the patient population [8]. Although the actual donation rates are unknown, as ethnicity is not registered, the prevalence of the extended blood groups in the donor pool suggests there is an underrepresentation [9].

Past studies examining donation barriers and motivators suggest a lower donation awareness and more negative donation attitudes towards donating blood in potential African donors [1, 10]. For example, Lemmens, Abraham [11] found a significant positive correlation between knowledge and affective and cognitive attitude. However, knowledge and awareness are related but not the same concepts [12]. As past research mainly gave descriptive findings of self‐reported barriers and motivators and often in one group, there is little evidence in the blood donation context on how awareness and attitudes relate and differ for ethnic groups. Our aim was to focus more in depth on this topic by studying the association with and differences between blood donation awareness and blood donation attitudes for people of Dutch and African descent who had no former blood donation experiences in the Netherlands.

### Theoretical background

Unawareness has been consistently identified as a main blood donation barrier for non‐donors [13]. Unawareness of blood donation can be defined as unconsciousness, which can be caused by never having heard of or communicated about blood donation [14, 15] or never having taken in and remembered information about it [16]. It can be expressed in practical unfamiliarity such as not knowing the name or the location of the blood bank organization or not knowing individuals who have donated blood and not having talked about this subject [14, 17].

Awareness is an important factor in the transtheoretical model [18]. This model comprises of five stages of change: Precontemplation, Contemplation, Preparation, Action and Maintenance. The Precontemplation stage is characterized by unawareness, and individuals within this stage do not plan to change behaviour in the near future because they have not yet contemplated about it [19]. African minorities and migrants generally seem to be less aware of blood donation than the White majority group [1]. For instance, Burditt, Robbins [19] found that a majority of the African American participants were in the Precontemplation stage [18], indicating a low awareness. Unawareness of blood donation has been reported as a major reason why African minority groups are underrepresented as blood donors, while on the other hand these groups seem relatively more motivated by awareness raising and donation requests than White majority groups [1]. In an interview study among Ghanaians and African‐Surinamese people in the Netherlands, only 11% of the participants recognized the name or logo of the Dutch blood bank organization: ‘Sanquin’ [10]. Participants had not actively thought of becoming a blood donor, also because they had never been approached about it. These findings correspond with a study in Australia, where African participants reported that they had never discussed blood donation before the study [14].

Besides a practical unfamiliarity of blood donation (i.e. the name of the blood bank, what donating blood entails), unawareness can also be expressed in cognitive and affective unfamiliarity, such as not being aware of the importance of donating blood or overestimating the disadvantages of donating blood [13, 20].

Attitudes are described in the Theory of Planned Behaviour as how positive or negative a specific behaviour is evaluated by individuals and can be divided into two sub components; cognitive attitude and affective attitude. The first comprises the evaluative judgements to the behaviour, while the later comprises emotional reactions to the behaviour [21, 22]. Attitudes have been found to be important determinants of intention and, respectively, behaviour [22, 23, 24, 25] and seem to play an important part particularly in the Precontemplation stage in behaviour change [18, 26]. There is evidence that African minorities have a slightly more negative attitude towards blood donation compared to White majority populations in Western countries [1]. Various studies in the United States showed that African Americans less often believe it is important to donate blood (e.g. because of the perception that their blood is not wanted or will be wasted) and that they are generally more afraid of needles and pain as compared to White Americans [1, 27, 28]. In our qualitative interview study among African migrants living in the Netherlands, the overall general opinion towards giving blood was positive, but participants reacted more hesitantly when asked about their own attitude to donate blood [10].

It is expected that it could be beneficial to design recruitment campaigns and their target group with the knowledge from the TTM and TPB in mind. Based on these models and the literature, we expect that an assessment of the level of and interplay between awareness and attitudes in our potential donor recruitment target group will benefit the development of future donor recruitment strategies. Partly due to being unaware, individuals with low awareness or in the Precontemplation stage may experience resistance to behaviour change or believe the costs of behaviour change are higher than the benefits [26]. Therefore, when the target audience becomes well informed, the gained awareness might alleviate cognitive and affective unfamiliarity surrounding blood donation and act as a prerequisite for positive attitude change and a move to the next step in the TTM.

Based on the studies and mechanisms discussed above, the following hypotheses were developed:

H1: African ethnic minorities have a lower blood donation awareness compared with people of Dutch descent in the Netherlands.

H2: African ethnic minorities have a lower blood donation attitude compared with people of Dutch descent in the Netherlands.

H3: A higher blood donation awareness is associated with higher blood donation attitudes for African ethnic minorities and people of Dutch descent.

## Methods

### Design and participants

For this study, we made use of various survey data. The Motivations to Give, Donate and Share study (Motive‐study; https://www.sanquin.org/research/donor‐studies‐projects/motive‐study/index) consists of stratified online survey data collected in 2018 among people of African background and university students, residing in the Netherlands [29, 30]. Participants in the Motive‐study were recruited among the general population through an ISO‐certified research company (Panelclix) and among students through Tilburg University. Participants recruited through Panelclix were compensated with ‘Clix’, which can be traded for a small amount of money. Participants recruited through the university were compensated with course credits. Next to data originating from the Motive‐study, additional data were also collected in 2018 among social media users (regardless of ethnic background) using a shortened version of the survey used in the Motive‐study. An overview of the recruitment of the samples can found in Table 1. Adequate Dutch or English language proficiency was a requirement for participation. Social media users were recruited predominantly via Facebook and WhatsApp, using convenience‐ and snowball‐sampling. The surveys were shared on the social media account of the Dutch blood bank organization Sanquin and also via Promoted Posts to reach social media users who do not ‘like’ or follow this Facebook page. Participants recruited through social media were compensated with a voucher for a large online store. To our knowledge, no recruitment campaigns aimed at the target group that could interfere with the results were rolled out at the times of data collection.

**Table 1:** Overview of survey data incorporated in current study

Study	Motive‐sample		Additional sample
Type of sample	African background	University students	Social media users
1. Data collection period	April–September 2018	April–May 2018	March–April 2018
2. Time to complete survey	30 min	30 min	10 min
3. Type of survey	Online, Qualtrics	Online, Qualtrics	Online, Qualtrics
4. Eligible age range	18–65 years	18–65 years	18 years and older
5. Eligible ethnic background	Only Sub‐Saharan African, African‐Surinamese and African‐Caribbean	All ethnicities	All ethnicities
6. Residing in the Netherlands	Yes	Yes	Yes
7. Recruitment	Panelclix and social media	Psychology lab Tilburg University	Social media
8. Language	Dutch & English	Dutch & English	Dutch
9. Total number of participants	*n* = 300	*n* = 141	*n* = 231
10. Participants included in current study	*n* = 248	*n* = 64	*n* = 97
11. Included participants of African descent	*n* = 248	*n* = 7	*n* = 2

For the analyses in the present study, we selected participants between the ages of 18 and 65, being the age range individuals can register as a blood donor in the Netherlands. We only included participants who had never donated blood but were not definitely excluded for blood donation and who were either of Dutch or African ethnic background. In total of the initial 672 participants combined, 3 (0·4%) were excluded for being either too young or too old for this study’s purpose, 65 (9·7%) were excluded for being of different ethnic background than African or Dutch, 133 (19·8%) for having donated blood previously, 23 (3·4%) for being definitely excluded to donate blood and 39 (5·8%) because of missing values on at least one of the variables included in the analyses of this study. The final sample included 257 people with a Sub‐Saharan, Afro‐Surinamese or Afro‐Caribbean (African) background (63%) and 152 people of Dutch background (37%).

Ethical approval for the study was granted by the Ethical Advisory Board of Sanquin and the Ethics Committee of Tilburg University. The project protocol this study is part of was also reviewed by the Medical Ethics Review Committee of the Academic Medical Center Amsterdam (now Amsterdam UMC – location AMC), but was waived from requiring medical ethical approval because the protocol did not fall under the Medical Research Involving Human Subjects Act of the Dutch law.

### Measures

#### Ethnicity and background characteristics

African background in this study refers to individuals of sub‐Saharan descent, which is defined as individuals who reported they had at least one parent originating from sub‐Sahara Africa, Surinam or the Caribbean and who indicated they were of sub‐Saharan, African‐Surinamese or African‐Caribbean descent. Dutch background was defined as individuals who reported that both parents are Dutch and who were born in the Netherlands. Additionally, individuals who were born abroad and whose parents were born abroad were defined as first‐generation migrants, and individuals who were born in the Netherlands with at least one parent who was born abroad were defined as second‐generation migrants [31]. Sociodemographic background variables were age, gender and educational level. Educational level was measured using the International Standard Classification of Education (ISCED) adjusted version to the Dutch educational system of Statistics Netherlands [32, 33]. The seven answer categories were then divided in low (no education till lower secondary education), middle (upper secondary and post‐secondary non‐tertiary education) and high (Bachelor’s degree and higher) educational levels.

#### Donation awareness

Donation awareness was measured with two separate questions. ‘Have you ever heard of the Dutch blood bank organization, Sanquin?’ and ‘Do you personally know someone who has donated blood or is currently a blood donor?’ with ‘yes’ (a score of 1) and ‘no’ (a score of 0) as the possible answer categories (*r *= 0·30, *p* < 0·001). Both questions were combined to assess the participants’ level of awareness with a score of 0 ‘no awareness’, 1 ‘low awareness’ (knows either the blood bank or a blood donor) or 2 ‘high awareness’ (knows both the blood bank and a blood donor). The first awareness question had the follow‐up question ‘How have you heard of Sanquin? You can select multiple options’ with various peers and media as sources (i.e. through family members, through social media) as answer categories. The second awareness question was followed up by the question ‘Who has donated blood or is currently a blood donor that you know of? You can select multiple persons.’ with various peers presented as options (i.e. colleagues/fellow students, relatives). Both follow‐up questions had an ‘other, namely …’ option.

#### Donation attitudes

Donation attitudes were measured using six questions with a 7‐point bipolar statements, based on the measures validated by France, Kowalsky [26]. The time frame was adjusted from eight weeks in the original scale to 12 months, as the blood donation procedure in the Netherlands is more lengthy compared to donating at the blood drives in the United States (e.g. you cannot donate at your first visit) and a blood donor in the Netherlands donates on average only once or twice annually [29]. A factor analysis verified that donation attitudes could be divided into cognitive attitude (evaluative judgements towards blood donation) (α = 0·915) and affective attitude (emotional reactions towards blood donation) (α = 0·863), which both accounted for 75·0% of the total variance in the six items. When analysed separately for participants of African and Dutch background, exactly the same two factors arose and the Cronbach alphas were still good (cognitive attitude: α = 0·927 African background, α = 0·852 Dutch background; affective attitude: α = 0·888 African background, α = 0·821 Dutch background). Both cognitive attitude (useless/useful, pointless/worthwhile and the wrong thing to do/the right thing to do) and affective attitude (unpleasant/pleasant, unenjoyable/enjoyable, frightening/not frightening) are constructed from the mean of three 7‐point bipolar statements. On both scales, a score of 0 refers to the lowest possible attitude, while a score of 6 refers to the highest possible attitude.

### Analyses

Statistical software (SPSS, version 23, Chicago, IL) was used to examine the descriptive properties of the data and test our hypotheses. *t*‐Tests and chi‐square tests were performed to test differences in demographic characteristics between the two ethnic groups. The differences in donation awareness and donation attitude between participants of African and Dutch ethnic background were tested using logistic and linear regression analyses and controlled for age, gender and educational level to find answers for hypotheses H1 and H2. Multivariate linear regression analyses tested the associations of donation awareness on cognitive and affective attitude. We made two separate (stratified) models for those of African and Dutch descent (H3). These sub‐group analyses were controlled for age, gender and educational level, plus migrant generation in the African sub‐sample.

## Results

### Background characteristics of samples and Dutch and African individuals

Table 2 shows the sociodemographic background characteristics of the 152 participants of Dutch and the 257 participants of African descent. There were more women than men in both groups, namely 63% (*n* = 96) in the Dutch group and 72% (*n* = 184) in the African group, but the difference between the two ethnic groups was not significant (*X^2^*(1) = 3·1, p = 0·08). The overall age was relatively lower in the Dutch background group (*M* = 25·7, *SD* = 10·5) compared with the African group (*M* = 35·9, *SD* = 12·5; *t* (361·8) = −8·9, p < 0·001). Also, the Dutch group was significantly higher educated compared with the African group (*X^2^*(2) = 35·8, p < 0·001). Among the African individuals, 48% (*n* = 124) were first‐generation migrants and 52% (*n* = 133) were second‐generation migrants (see Appendix A for the demographic comparison of the three study samples).

**Table 2:** Background characteristics for Dutch and African individuals (N = 409)

Characteristic	Dutch background (*n* = 152)	African background (*n* = 257)	Significance testing
	*n (%)/M (SD)*	*n (%)/M (SD)*	*t (df)/X^2^ (df)*
Gender			*X^2^(*1*)* = 3·1
Male	56 (37%)	73 (28%)	
Female	96 (63%)	184 (72%)	
Age	25·7 (10·5)	35·9 (12·5)	*t (*361·8*) *= −8·9^***^
Educational level			*X^2^(*2*)* = 35·8^***^
Low	6 (4%)	27 (11%)	
Middle	37 (24%)	124 (48%)	
High	109 (72%)	106 (41%)	
Migrant generation			
First	n.a.	124 (48%)	
Second	n.a.	133 (52%)	

^***^ p < 0·001.

### Differences in donation awareness and donation attitudes

**Figure 1:**
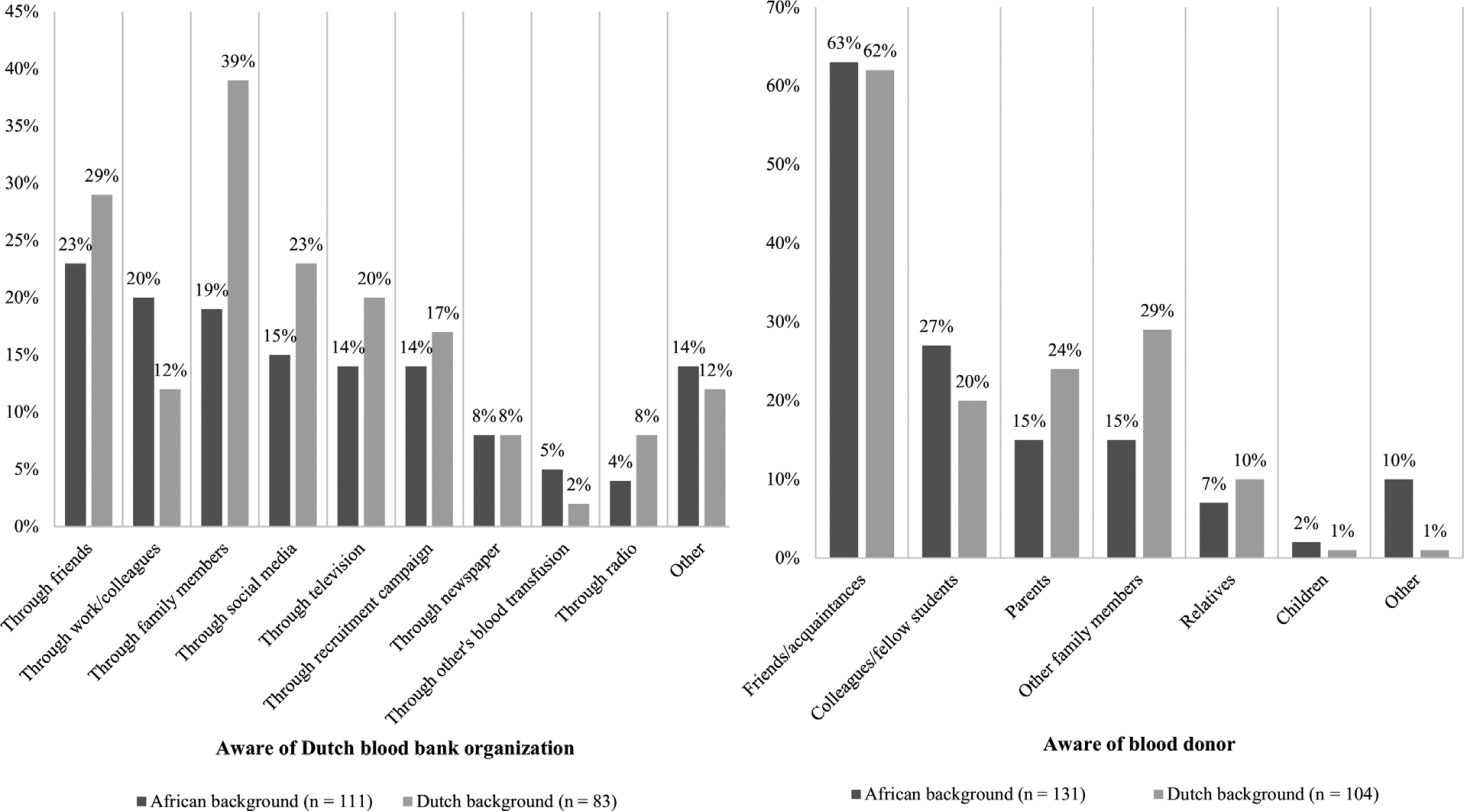
Specified blood bank awareness (*n* = 194) and knowing another donor (*n* = 235), divided by ethnic background.

Slightly more than half of the Dutch individuals knew Sanquin (55%; *n* = 83) (Table 3). This percentage was significantly lower among the African individuals (43%; *n* = 111) (adjusted odds ratio; *AOR* = 0·63, 95% CI [0·40, 0·99], *p* < 0·05). Of those participants who were familiar with Sanquin (*n* = 194), the Dutch participants (*n* = 83) most often heard of it through family members (39%; *n* = 32) and the African participants (*n* = 111) most often heard of it through friends (23%; *n* = 26) (Figure 1). A relatively large part of the African individuals also became familiar with the blood bank through work and/or colleagues (20%; *n* = 22). In the ‘Other’ category, individuals of Dutch background most often mentioned that knowing Sanquin is part of their general knowledge, whereas individuals of African background most often mentioned they knew Sanquin via the Internet. More than two‐thirds of the Dutch individuals knew someone personally who has donated blood (68%; *n* = 104), whereas about half of the African individuals mentioned this (51%; *n* = 131) (*AOR* = 0·56, 95% CI [0·35, 0·90], p < ·05). Of those participants who personally knew one or more other blood donors (*n* = 235), this was in most cases a friend or acquaintance (63% (*n* = 66) of the Dutch individuals and 62% (*n* = 81) of the African individuals) (Figure 1). In the ‘Other’ category, individuals of African background mentioned for instance parents in law, the partner and a neighbour. When combining the two donation awareness measures, we notice that relatively most individuals of Dutch background belong to the ‘high awareness’ category (45%, *n* = 70), while relatively least individuals of African background fit in this category (28%, *n* = 72). H1 can be accepted as less African individuals were aware than Dutch individuals.

**Table 3:** Differences in awareness and attitudes between Dutch and African individuals (N = 409)

	Dutch background (*n* = 152)	African background (*n* = 257)	B/AOR
Aware blood bank	*n (%)/M (SD)*	*n (%)/M (SD)*	*AOR* = 0·63*
Yes	83 (55%)	111 (43%)	
No	69 (45%)	146 (57%)	
Aware other donor			*AOR* = 0·56*
Yes	104 (68%)	131 (51%)	
No	48 (32%)	126 (49%)	
Awareness combined			
Not aware	34 (22%)	88 (35%)	
Low awareness	51 (33%)	94 (37%)	
High awareness	70 (45%)	72 (28%)	
Cognitive attitude	4·7 (1·2)	3·9 (1·7)	*B* = −0·90***
Affective attitude	2·7 (1·4)	2·9 (1·5)	*B* = 0·10

All tests are adjusted for gender, age and educational level. Dutch individuals were the reference group.

^*^ p < 0·05.

^***^ p < 0·001.

Dutch individuals showed an overall higher cognitive attitude (*M* = 4·7, *SD* = 1·2) compared with African individuals (*M* = 3·9, *SD* = 1·7). After controlling for age, gender and educational level, this difference remained significant (*B *= −0·90, *t*(403) = −5·17, p < 0·001). Regarding affective attitude, we found no significant differences between the Dutch (*M* = 2·7, *SD* = 1·4) and African individuals (*M* = 2·9, *SD* = 1·5; *B* = 0·10, *t*(403) = 0·59, p = 0·55). Thus, H2 could only be partly accepted as there was a significant difference in cognitive attitude, but not in affective attitude.

### The associations between donation awareness on donation attitudes

Table 4 summarizes the results for the multivariate linear regression analyses on donation attitudes. Among the African individuals, high awareness was significantly associated with a higher cognitive attitude (*B* = 0·98, *t*(249) = 3·61, p < 0·001), whereas this was not the case for Dutch individuals who showed no significant association between awareness and cognitive attitude (*B* = 0·36, *t*(145) = 1·49, p* *= 0·14).

**Table 4:** Multivariate linear regressions on cognitive and affective attitude for individuals of Dutch (*n* = 152) and African background (*n* = 257)

*Variables*	Cognitive attitude				Affective attitude			
	Dutch		African		Dutch		African	
	*B (SE)*	*t*	*B(SE)*	*t*	*B(SE)*	*t*	*B(SE)*	*t*
Intercept	4·57 (0·56)	8·14	3·77 (0·54)	7·02	3·14 (0·72)	4·35	3·03(0·50)	6·12
Blood donation awareness								
Not aware	Ref.		Ref.		Ref.		Ref.	
Low awareness (knows either the blood bank or blood donor)	0·31 (0·25)	1·25	0·28 (0·25)	1·11	0·36 (0·32)	1·10	0·08(0·23)	0·33
High awareness (knows both the blood bank and blood donor)	0·36 (0·24)	1·49	**0**·**98 (0**·**27)** ***	**3**·**61**	0·17 (0·31)	0·54	**0**·**56(0**·**25)** *	**2**·**23**
Female	**0**·**65 (0**·**19)** ***	**3**·**43**	−0·01 (0·24)	−0·03	−0·02 (0·24)	−0·08	−0·02(0·22)	−0·07
Age in years	0·01 (0·01)	0·79	−0·01 (0·01)	−0·74	0·004 (0·01)	0·32	0·01(0·01)	0·84
Second migrant generation	n.a.		−0·11 (0·23)	−0·48	n.a.		−0·16(0·21)	−0·76
Educational level								
Low	Ref.		Ref.		Ref.		Ref.	
Middle	−0·45 (0·50)	−0·90	−0·02 (0·35)	−0·06	−0·81 (0·64)	−1·27	−0·55(0·33)	−1·69
High	−0·85 (0·48)	−1·79	0·03 (0·37)	0·07	−0·78 (0·61)	−1·26	−0·60(0·34)	−1·79

Bold values are statistically significant.

^*^ p < 0·05.

^***^ p < 0·001.

Regarding the models for affective attitude, scoring high on awareness was significantly associated with a higher affective attitude among the African background group (*B* = 0·56, *t*(249) = 2·23, p < 0·05), but not in the Dutch background group (*B* = 0·17, *t*(145) = 1·10, p = ·59).

Based on our results, H3 was accepted for the African individuals as a higher awareness was associated with higher cognitive and affective attitudes, but rejected for the Dutch individuals.

## Discussion

In this study, we hypothesized that African migrants have a lower blood donation awareness and a more negative attitude towards donating blood than Dutch individuals and that a higher donation awareness is associated with a higher donation attitude. In line with our first hypothesis and the literature, we found that less African individuals are aware than the Dutch individuals. However, whereas only 11% of African migrants was aware of the Dutch blood bank organization in our previous, qualitative study sample [10], awareness in this current sample was much higher at 43%. This might be due to a difference in the composition and recruitment strategies of the study samples. For example, in the present study sample a much larger part of the participants are second‐generation migrants. Additionally, they were recruited online and the participants with an African background most often mentioned they knew Sanquin via the Internet.

Similar to previous studies and partly in concordance with our second hypothesis, participants with an African background had a lower cognitive, but not a lower affective, attitude than Dutch individuals. This is partly in line with previous studies, African migrants and minorities generally report a lower attitude towards blood donation than the majority population [10, 14]. No other studies that we are aware of studied cognitive and affective blood donation attitudes separately for different ethnic groups.

Lastly, we hypothesized that awareness and attitudes would be positively correlated for all individuals. Interestingly, we only found a positive association between awareness and attitudes in the African group. Perhaps other deterrents or motivators, such as self‐efficacy or (in)convenience, might be more impactful for donation attitudes in this group [34, 35].

As this study highlights that more people with an African background may be in the ‘precontemplation stage’ of the TTM than people with a Dutch background, it could be beneficial for Sanquin to design future recruitment campaigns and target strategies such that they can help African individuals to move to the contemplation or even preparation stage [18, 23, 24]. As awareness seems to increase attitudes, this might eventually lead to behaviour change according to the TPB [23].

The current study adds to the literature regarding this topic with insights from a European context, which differs historically, culturally and socio‐economically from the United States or Australia, where most research is conducted [1]. However, a limitation of this study is the recruitment of convenience samples of through a panel, a university and social media. As the two ethnic groups are unequally represented in these contexts, the Dutch sample is of younger age and more highly educated, which also makes it less generalizable to the Dutch general population. Another consideration is that the surveys were administered in English and Dutch only and also distributed online, which can be an obstacle for first‐generation African migrants. We made this choice because Dutch or English fluency is required to donate blood in the Netherlands. Additionally, the blood donor registration process is also online, thus basic Internet competencies are essential for blood donors. The current sample might not be generalizable to all African migrants living in the Netherlands, but is generalizable to the potential donor population of African descent.

The results of this study have valuable implications for future research and blood donor recruitment. Awareness raising might be a promising first step to help African minorities contemplating about blood donation. In the Netherlands, Sanquin is the only blood bank organization. If a person does not recognize the name or logo, he or she will not notice a blood collection centre nearby and it also makes it difficult to register as a donor, as the person does not know where to do so. We did find that a higher level of awareness – both being familiar with the blood bank organization and personally knowing a blood donor – to have a positive association with attitudes. The importance of a social network needs to be highlighted in this, as most individuals became familiar of blood donation through family members and friends, as also was emphasized in other studies [36, 37]. Since ethnic minorities often have less blood donors in their social network, it is of importance to reach the target communities first as to enable blood donation awareness to spread through their social networks [9, 10, 37]. This is essential information for the development of future blood donor recruitment interventions.

## Conflict of interest

The authors declare that they have no competing interests.

## Data Availability

Data of the Motive‐study or the particular data set used for this study are available upon request by contacting Elisabeth Klinkenberg (L.Klinkenberg@Sanquin.nl). More information on the Motive‐study can be found here: https://www.sanquin.org/research/donor‐studies‐projects/motive‐study/index

## Appendix A

### Background characteristics for the survey data incorporated in current study

**Table:**

Study	Part of Motive‐study		Separate study	Significance testing
	African background (*n* = 248)	University students (*n* = 64)	Social media users (*n* = 97)	
Characteristic	*n (%)/M (SD)*	*n (%)/M (SD)*	*n (%)/M (SD)*	*F (df)/X^2^ (df)*
Gender				*X^2^(*2*) =* 3.5
*Male*	70 (28%)	22 (34%)	37 (38%)	
*Female*	178 (72%)	42 (66%)	60 (62%)	
Age	36.4 (12.4)	20.6 (2.5)	28.7 (12.0)	*F*(2, 406) = 55.4***
Educational level				*X^2^(4) = 76.5* ***
*Low*	27 (11%)	0 (0%)	6 (6%)	
*Middle*	124 (50%)	0 (0%)	37 (38%)	
*High*	97 (39%)	64 (100%)	54 (56%)	
Ethnic background				*X^2^(2) = 373.9* ***
*Dutch*	0 (0%)	57 (89%)	95 (98%)	
*African*	248 (100%)	7 (11%)	2 (2%)	

* p < 0.05.

** p < 0.01.

*** p < 0.001.
